# Birth weight and blood lipid levels in Spanish adolescents: Influence of selected APOE, APOC3 and PPARgamma2 gene polymorphisms. The AVENA Study

**DOI:** 10.1186/1471-2350-9-98

**Published:** 2008-11-10

**Authors:** Jonatan R Ruiz, Idoia Labayen, Francisco B Ortega, Luis A Moreno, Domingo González-Lamuño, Amelia Martí, Esther Nova, Miguel García Fuentes, Carlos Redondo-Figuero, J Alfredo Martínez, Michael Sjöström, Manuel J Castillo, the AVENA Study Group

**Affiliations:** 1 Department of Medical Physiology, School of Medicine, University of Granada, 18071 Granada, Spain; 2 Unit for Preventive Nutrition, Department of Biosciences and Nutrition at NOVUM, Karolinska Institutet, Huddinge, Sweden; 3 Department of Nutrition and Food Science, University of the Basque Country, Vitoria, Spain; 4 E. U. Ciencias de la Salud, University of Zaragoza, Zaragoza, Spain; 5 Department of Pediatrics, University of Cantabria, Santander, Spain; 6 Department of Physiology and Nutrition, University of Navarra, Pamplona, Spain; 7 Immunonutrition Research Group, Department of Metabolism and Nutrition. Consejo Superior de Investigaciones Científicas, Madrid, [E.N.], Spain

## Abstract

**Background:**

There is increasing evidence indicating that genes involved in certain metabolic processes of cardiovascular diseases may be of particular influence in people with low body weight at birth. We examined whether the apolipoprotein (APO) E, APOC3 and the peroxisome proliferator-activated receptor-γ-2 (PPARγ2) polymorphisms influence the association between low birth weight and blood lipid levels in healthy adolescents aged 13–18.5 years.

**Methods:**

A cross-sectional study of 502 Spanish adolescents born at term was conducted. Total (TC) and high density lipoprotein cholesterol (HDLc), triglycerides (TG), apolipoprotein (apo) A and B, and lipoprotein(a) [Lp(a)] were measured. Low density lipoprotein cholesterol (LDLc), TC-HDLc, TC/HDLc and apoB/apoA were calculated.

**Results:**

Low birth weight was associated with higher levels of TC, LDLc, apoB, Lp(a), TC-HDLc, TC/HDLc and apoB/apoA in males with the APOE ε3ε4 genotype, whereas in females, it was associated with lower HDLc and higher TG levels. In males with the APOC3 S1/S2 genotype, low birth weight was associated with lower apoA and higher Lp(a), yet this association was not observed in females. There were no associations between low birth weight and blood lipids in any of the PPARγ2 genotypes.

**Conclusion:**

The results indicate that low birth weight has a deleterious influence on lipid profile particularly in adolescents with the APOE ε3/ε4 genotype. These findings suggest that intrauterine environment interact with the genetic background affecting the lipid profile in later life.

## Background

There is increasing evidence indicating that low birth weight is a risk factor for cardiovascular disease. Indeed, among persons born at term, those with lower birth weights have a higher relative risk of metabolic health problems in early adulthood, including hypertension, cardiovascular disease, and type 2 diabetes [1]. In this context, findings from the AVENA Study (*Alimentación y Valoración del Estado Nutricional en Adolescentes*, Food and Nutritional Status in Spanish Adolescents) indicated that birth weight is associated with a number of cardiovascular disease risk factors in adolescents such as total and central body fat, fat free mass, and muscular fitness [2-5].

One of the hypotheses explaining this phenomenon is the predisposition to adopt a "thrifty phenotype". According to this theory, fetal adaptation to an adverse intrauterine environment involves programming of pathways that might predispose to metabolic abnormalities and cardiovascular disease in later life. The intra-uterine under- or over-nutrition can alter the gene expression of the fetus, causing developmental adaptations that may lead to permanent changes in physiology and metabolism; changes that may have consequences later in life.

Findings of several cohort studies indicate that low birth weight is associated with adverse plasma lipid profile [6-8], whereas others have suggested that this association is too small to be considered of public health importance and that other variables as postnatal weight gain could act as confounder of this relationship [9]. One would predict that genes involved in the metabolic processes of these diseases may have different effects on people with different body weight at birth. This phenomenon has been described in relation to blood lipids and the apolipoprotein (APO) E gene [10], and both blood lipids and insulin resistance and the peroxisome proliferator-activated receptor-γ-2 (PPARγ2) gene [11,12]. We have studied the effect of the Ala12 allele in the PPARγ2 gene on the relationship between birth weight and body composition in Spanish adolescents, and we showed that small body weight at birth may program lower fat free mass in adolescents carrying the Ala12 allele [13].

Coronary heart disease is a leading cause of global mortality. The relationship between blood lipids and the development of coronary heart disease in children and adolescents is well established. Results from the AVENA Study showed that 20–30% of Spanish adolescents present an unfavorable blood lipid profile [14,15]. In the AVENA study, the APOE, APOC3 and PPARγ2 gene polymorphisms were genotyped. The ApoE gene is one of the most important genetic determinants of atherogenesis, since its major function is to regulate the hepatic clearance of triglycerides (TG) rich particles such as chylomicron remnants and very low density lipoprotein remnants. The APOC3 gene have also been proposed as being potentially responsible for the occurrence of lipid profile disturbances, since it has an inhibitory effect on lipoprotein lipase activity and hepatic uptake of lipoproteins [16]. Finally, the PPARγ-2 has been shown to enhance lipoprotein TG hydrolysis by endothelial lipoprotein lipase, which in turn seems to affect lipid profile [17].

To understand whether an adverse intrauterine environment may alter the expression of these cardiovascular disease-related genes is clinically relevant. This study aims to clarify to what extent variants of these genes might interact with birth weight in determining the blood lipid profile later in life. Therefore, we examined the influence of APOE, APOC3 and PPARγ2 gene polymorphisms on the association between low birth weight and blood lipid levels in Spanish adolescents from the AVENA Study.

## Methods

The AVENA Study is a cross-sectional study designed to assess the nutritional status of a representative sample of Spanish adolescents aged 13 to 18.5 years. Data collection took place from 2000 to 2002 in five Spanish cities (Madrid, Murcia, Granada, Santander and Zaragoza). The complete methodology of the study has been described in detail elsewhere [15,18]. The number of adolescents included in the AVENA Study was 2859 adolescents. Blood samples and DNA data were randomly obtained from 502 participants. The subgroup from which blood samples were obtained was similar to the remaining subjects regarding the variable selected to calculate the number of participants to be included in the study, i.e. body mass index (BMI) [15], as well as regarding age (P = 0.750) and gender proportions (P = 0.320). A comprehensive verbal description of the nature and purpose of the study was given to the adolescents, their parents and teachers. Written consent to participate was requested from both parents and adolescents. Adolescents with personal history of cardiovascular disease, under medication at the time of the study, or those who were pregnant, were excluded. The study protocol was performed in accordance with the ethical standards laid down in the 1961 Declaration of Helsinki (as revised in Hong-Kong in 1989, and in Edinburgh in 2000), and approved by the Review Committee for Research Involving Human Subjects of the Hospital Universitario Marqués de Valdecilla (Santander, Spain).

Before any testing was performed, the parents completed a questionnaire, part of which addressed the adolescents' previous and current health status. Socioeconomic status was also assessed via the questionnaire, and was defined by the educational level and occupation of the father. According to this information, and following the recommendation of the Spanish Society for Epidemiology, the adolescents were classified into five categories: low (I), medium-low (II), medium (III), medium-high (IV) and high socioeconomic status (V).

### Neonatal data

Data on birth weight and gestational age at birth were obtained from health booklets records that are issued at birth and where child's paediatricians records birth weight, charts the infant's growth and vaccinations. Birth weight was expressed as the standard deviation from the expected weight calculated with the use of reference standards previously described for this population, according to sex and gestational age [19]. This variable (called birth weight score) will be used for the analyses.

Gestational age was coded as 1 for those who were born between the 35^th ^to 40^th ^week of gestation, and 2 for those who were born after the 40^th ^week of gestation. According to this information, 80.9% and 77.0% of males and females, respectively, were born between 35 to 40 weeks of gestation, and 12.2% of males and 15.1% of females were born after more than 40 weeks of gestation. The percentage of adolescents born before 35 weeks of gestation was 6.9% of the males and 7.9% of the females. This group was not included in the analyses.

### Physical examination

Anthropometric measurements were obtained as described elsewhere [20,21]. BMI was calculated as weight in kilograms divided by square of height in meters (kg/m^2^). Skinfold thickness was measured at the biceps, triceps, subscapular, suprailiac, thigh and calf on the left side of the body to the nearest 0.2 mm using a Holtain skinfold caliper. Body fat percentage was calculated from skinfold thicknesses (triceps and subscapular) using Slaughter's equations [22], and fat free mass (kg) was derived by subtracting fat mass from total body weight. Reference values for anthropometric measurements of the AVENA Study can be found elsewhere [20,21].

Identification of pubertal development was assessed according to Tanner & Whitehouse [23]. Self-reported genital development in males and breast development in females were used for pubertal stage classification.

### Cardiorespiratory fitness

Cardiorespiratory fitness was assessed by the 20 m shuttle run test as previously described [24]. It was considered as the number of stages completed (precision of 0.5 steps). All participants were familiarized with the test, since the 20 m shuttle run test is one of the fitness tests included in the physical education curriculum in our country. Reference values of fitness levels have been reported for the whole study population as well as for those adolescents from whom blood sample was obtained [25].

### Blood sampling

Blood (20 ml) was collected from an antecubital vein between 8:00 and 9:00 a.m, after an overnight fast. Serum levels of total (TC) and high density lipoprotein cholesterol (HDLc), as well as the levels of TG, apolipoprotein (apo)A and B, and lipoprotein(a) [Lp(a)] were measured. The coefficients of variation were less than 3% and the intra-class coefficients were higher than 0.96% for all blood variables. Quality control of the assays was assured by the Regional Health Authority, as is compulsory for all hospital clinical laboratories in Spain.

Low density lipoprotein cholesterol (LDLc) was calculated with the Friedewald formula [26]. The following atherogenic indices were also calculated: TC-HDLc, TC/HDLc, and apoB/apoA. A detailed description of the blood analysis as well as reference values for lipid and lipoprotein has been reported by Ruiz et al. [15,27].

### Genotyping

Genomic DNA was extracted and purified from 500 μL of whole blood treated with EDTA, using the Quiagen procedure described by Higuchi [28]. APOE genotypes were determined by polymerase chain reaction (PCR) and allele-specific restriction digestion of the amplified products with the restriction enzyme HhaI, as described elsewhere [29] (rs7412, and rs429358). APOE genotypes were encoded as 1 = ε2/ε3, 2 = ε3/ε3, and 3 = ε3/ε4. APOC3 genotypes were determined by PCR and allele-specific restriction digestion of the amplified products with the restriction enzyme SstI following the procedures described elsewhere [30]. APOC3 genotypes were encoded as 1 = S1/S1 and 2 = S1/S2 (rs5128). The PPARγ2 genotypes were determined by the PCR method and further digestion of products with *Bst*U-I restriction enzymes as previously described [31], and were encoded as 1 = Pro12/Pro, and 2 = Pro12/Ala and Ala12/Ala genotype [32] (rs1801282). The percentage of adolescents with the Ala12/Ala genotype was 1.3%, therefore this sub-group was analyzed together with the Pro12/Ala.

### Statistical analysis

Following a bivariate correlation analysis, multiple regressions were used to study the association between birth weight and blood lipid levels, for males and females separately (unadjusted model). Further analyses were done after controlling for age, pubertal stage, socioeconomic status, gestational age, BMI and cardiorespiratory fitness (adjusted model). A separate regression model was performed for each lipid parameter. Additional analyses were also performed by genotypes.

To stabilize variability and to achieve normality in the residuals, TC, HDLc, LDLc, TG, apoA, apoB and Lp(a) were transformed to the natural logarithm.

The impact on blood lipid levels of APOE, APOC3 and PPARγ2 genotypes was analyzed by one-way analysis of variance (ANOVA), for males and females separately. For the APOE genotypes, the subgroup means were compared by Tukey's test. Comparisons were adjusted for mass significance as described by Holm [33,34]. The method of Holm proceeds as follows: Sort the P-values of the k tests in increasing order, P_1_, P_2_, ..., P_i_, ..., P_k_. If P_1 _> α/k; none of the k tests are significant, and the test procedure is finished. If P_1 _≤ α/k, test 1 is significant, and now P_2 _is examined. If P_2 _> α/(k - 1), none of the (k - 1) remaining tests are significant, but if P_2 _≤ α/(k - 1), test 2 is significant and P_3 _is examined. This procedure goes on until P_i _> α/(k - i + 1), and the procedure is interrupted. This method keeps family error rate less than α. Family error rate is defined as the probability that one or more false significances out of k tests is less than or equal to α.

Interaction effects between birth weight and sex, and between birth weight and genotypes were tested by inserting product terms for the relevant variables. The analyses were performed using the Statistical Package for Social Sciences (SPSS, v. 15.0 for WINDOWS; SPSS Inc, Chicago) and the level of significance was set to 0.05.

## Results

*Descriptive characteristics of the study sample are shown in *Table 1.

**Table 1:** Descriptive characteristics of the study sample

	**Males (n = 260)**	**Females (n = 242)**
Age (years)	15.4 ± 1.4	15.4 ± 1.4
Tanner II,III,IV,V (%)	5.8, 15.1, 41.8, 37.3	1.6, 8.1, 54.3, 36.0
Weight (kg)	64.6 ± 13.4	56.8 ± 10.5
Height (cm)	170.7 ± 8.2	161.5 ± 6.4
Body mass index (kg/m^2^)	22.1 ± 3.9	21.7 ± 3.5
Cardiorespiratory fitness (stage)	7.0 ± 2.6	4.1 ± 1.8
Sum of 6 skinfolds	75.8 ± 38.1	98.8 ± 31.9
Percentage body fat	20.2 ± 10.4	25.6 ± 6.6
Triglycerides (mg/dl)	71.3 ± 31.6	64.7 ± 26.4
TC (mg/dl)	156.7 ± 26.4	169.9 ± 26.4
HDLc (mg/dl)	51.0 ± 9.7	59.2 ± 11.6
LDLc (mg/dl)	91.4 ± 23.9	97.4 ± 23.2
ApoA (mg/dl)	116.9 ± 17.1	127.7 ± 19.2
ApoB (mg/dl)	66.9 ± 14.3	69.9 ± 13.8
Lp(a) (mg/dl)*	14.1 ± 4.2	5.3 ± 3.9
ApoA/ApoB (mg/dl)	1.8 ± 0.5	1.9 ± 0.5
TC-HDLc (mg/dl)	105.7 ± 25.9	110.6 ± 25.8
Birth weight (g)	3443.0 ± 539.0	3283 ± 564
Birth weight score	409.0 ± 516.0	353 ± 559
Socieconomic status I,II,III,IV,V (%)	5.9, 25.3, 41.6, 23.6, 3.6	7.2, 25.4, 37.1, 23.3, 6.9

Values are means ± SD, otherwise indicated.

TC indicates total cholesterol; HDLc, high density liprotein cholesterol; LDLc, low density lipoprotein cholesterol; TG, triglycerides; Apo, apolipoprotein; Lp(a), lipoprotein a.

*Geometric mean ± SD.

### Interactions

There was a significant (all P < 0.05) interaction effect between birth weight and sex in all the outcome variables, therefore, all the analyses were performed separately for males and females. There was a significant interaction effect between birth weight and APOE in all of the outcome variables (TC, P = 0.02; HDL, P = 0.021; LDLc, P = 0.001; apoA, P = 0.049; apoB, P = 0.01; Lp(a), P = 0.051; TC-HDLc, P = 0.025; apoB/apoA, P = 0.054), except for TG (P = 0.362). There was also a significant interaction effect between birth weight and APOC3 (for TG, P = 0.025; apoA, P = 0.038, and Lp(a), P = 0.002). No significant (all P > 0.1) interaction effect between birth weight and PPARγ2 was found.

### Birth weight and lipids

**Figure 1:**
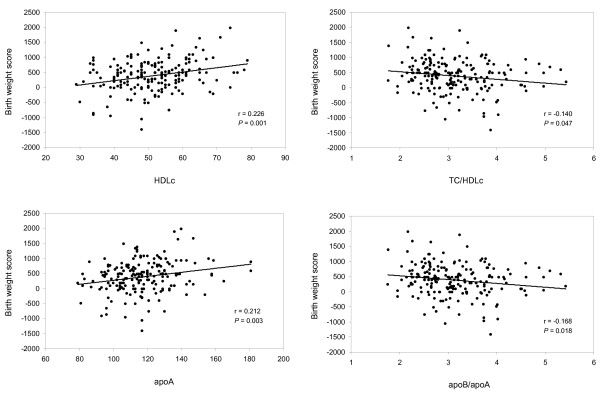
**Birth weight score (g) and blood lipid and lipoprotein levels (mg/dl) in male adolescents. **HDLc indicates high density lipoprotein cholesterol; apo, apolipoprotein; TC, total cholesterol.

In males, low birth weight was associated with lower levels of HDLc and apoA, and higher TC/HDLc and apoB/apoA ratios (Figure 1). Birth weight was not significantly associated with blood lipids in females. The results were similar after controlling for age, pubertal stage, socioeconomic status, gestational age, BMI and cardiorespiratory fitness (Table 2).

**Table 2:** Regression coefficients ($\hat{\beta }$) showing the association between birth weight score and blood lipid and lipoprotein levels by sex after controlling for age, pubertal stage, socioeconomic status, gestational age, body mass index and cardiorespiratory fitness

	**Males (n = 260)**		**Females (n = 242)**	
	
	$\hat{\beta }$	***P *value**	$\hat{\beta }$	***P *value**
TC*	0.035	0.682	0.073	0.476
HDLc*	0.134	**0.009**	0.058	0.537
LDLc*	-0.051	0.549	-0.044	0.668
TG*	-0.017	0.841	-0.06	0.541
apoA*	0.187	**<0.001**	-0.025	0.784
apoB*	-0.031	0.723	0.032	0.749
Lp(a)*	-0.085	0.334	0.051	0.614
TC-HDLc	-0.026	0.76	0.044	0.668
TC/HDLc	-0.066	**0.003**	-0.001	0.991
apoB/apoA	-0.113	**0.029**	0.049	0.609

TC indicates total cholesterol; HDLc, high density liprotein cholesterol; LDLc, low density lipoprotein cholesterol; TG, triglycerides; Apo, apolipoprotein; Lp(a), lipoprotein a.

*Analyses were performed on log transformed data.

### Genotypes and lipids

All the genotype frequencies of the analyzed gene polymorphism were in agreement with the Hardy-Weinberg equilibrium. The distribution of the APOE genotypes did not differ between males and females (P = 0.961) (Table 3). Males with the ε2ε3 genotype had lower levels of TC, LDLc and apoB compared with those with the ε3ε3 genotype. Similarly, females with the ε2ε3 genotype had lower levels of LDLc, and apoB compared with those with the ε3ε3 genotype. Females with ε2ε3 genotype had higher levels of apoA compared with those with the ε3ε3 genotype. There were no subjects with the ε2ε2, ε2ε4 or the ε4ε4 genotype.

**Table 3:** Lipid and lipoprotein levels (mg/dl), and body mass index (kg/m^2^) according to the APOE genotypes and sex

	**Males (n = 260)**				**Females (n = 242)**			
**Genotype**	**ε2/ε3**	**ε3/ε3**	**ε3/ε4**	**P value**	**ε2/ε3**	**ε3/ε3**	**ε3/ε4**	**P value**
**n (%)**	**22 (8.5)**	**186 (71.6)**	**52 (19.9)**		**21 (8.5)**	**175 (72.6)**	**46 (18.9)**	

TC**	144.2* ± 34.4	157.9 ± 24.8	160.6 ± 26.8	**<0.05**	160.8 ± 24.7	169.1 ± 25.1	177.0 ± 30.8	NS
HDLc**	53.0 ± 8.6	51.4 ± 9.6	49.8 ± 10.3	NS	65.5* ± 11.1	58.6 ± 11.0	58.5 ± 13.1	NS
LDLc**	77.2^† ^± 28.6	92.7 ± 22.9	95.3 ± 23.8	**<0.01**	83.2^† ^± 24.7	97.5 ± 21.9	103.4 ± 26.1	**<0.01**
TG**	69.7 ± 28.7	69.4 ± 28.1	77.5 ± 39.0	NS	60.4 ± 22.1	64.4 ± 26.1	68.0 ± 30.0	NS
apoA**	115.9 ± 15.6	118.1 ± 17.3	114.5 ± 16.3	NS	141.7^† ^± 15.6	125.7 ± 17.9	128.9 ± 23.3	**<0.01**
apoB**	55.6^† ^± 15.8	68.0 ± 13.0	68.8 ± 16.4	**<0.001**	59.3^† ^± 11.7	70.1 ± 12.9	73.9 ± 16.1	**<0.001**
Lp(a)**	23.1 ± 33.4	31.0 ± 35.1	33.1 ± 41.9	NS	14.3 ± 15.3	33.0 ± 40.3	37.6 ± 38.5	NS
TC-HDLc	91.1* ± 31.6	106.6 ± 24.3	110.8 ± 27.7	**<0.01**	95.3* ± 25.5	110.4 ± 24.0	118.5 ± 30.5	**<0.01**
TC/HDLc	2.7* ± 0.7	3.2 ± 0.7	3.4 ± 0.9	**<0.01**	2.5* ± 0.7	3.0 ± 0.6	3.1 ± 0.8	**<0.01**
apoB/apoA	0.5* ± 0.1	0.6 ± 0.1	0.6 ± 0.2	**<0.01**	0.4^† ^± 0.1	0.6 ± 0.1	0.6 ± 0.2	**<0.001**
BMI	20.9 ± 3.9	22.2 ± 3.8	22.4 ± 4.0	NS	21.5 ± 3.9	21.7 ± 3.6	21.8 ± 3.4	NS

Values are expressed as mean ± standard deviation, otherwise stated.

*P *value from ANOVA, adjusted for mass significance.

*P < 0.05; ^†^*P *< 0.01 for ε2/ε3 *vs *ε3/ε3 (Tukey test).

TC indicates total cholesterol; HDLc, high density liprotein cholesterol; LDLc, low density lipoprotein cholesterol; TG, triglycerides; Apo, apolipoprotein; Lp(a), lipoprotein (a); BMI, body mass index; NS, not significant.

**Analyses were performed on log transformed data, but non-transformed data are presented in the table.

The distribution of the APOC3 genotypes did not differ between males and females (P = 0.489) (Table 4). In males, levels of blood lipid were similar between S1/S1 and S1/S2 genotypes, whereas in females, adolescents with the S1/S1 genotype had lower levels of TC and TC-HCLc compared with those with the S1/S2 genotype.

**Table 4:** Lipid and lipoprotein levels (mg/dl) and body mass index (kg/m^2^) according to the APOC3 genotypes and sex

	**Males (n = 260)**			**Females (n = 242)**		
**Genotype**	**S1/S1**	**S1/S2**	**P value**	**S1/S1**	**S1/S2**	**P value**
**n (%)**	**214 (82.1)**	**46 (17.0)**		**198 (81.8)**	**44 (18.2)**	

TC*	156.2 ± 26.4	159.7 ± 27.0	NS	167.2 ± 25.2	181.9 ± 28.9	**<0.001**
HDLc*	51.0 ± 9.4	51.1 ± 11.4	NS	59.0 ± 11.4	60.0 ± 12.0	NS
LDLc*	91.1 ± 23.7	93.3 ± 25.2	NS	95.5 ± 22.4	106.3 ± 25.7	NS
TG*	70.5 ± 31.7	76.1 ± 31.3	NS	63.5 ± 26.1	70.6 ± 27.8	NS
apoA*	117.6 ± 17.2	114.1 ± 16.5	NS	127.0 ± 19.5	130.2 ± 18.1	NS
apoB*	66.6 ± 14.4	68.4 ± 14.0	NS	68.8 ± 13.1	74.9 ± 16.1	NS
Lp(a)*	31.2 ± 37.1	27.9 ± 32.4	NS	32.5 ± 39.4	31.8 ± 36.8	NS
TC-HDLc	105.2 ± 25.7	108.6 ± 27.0	NS	108.2 ± 23.9	121.8 ± 31.4	**<0.01**
TC/HDLc	3.2 ± 0.7	3.3 ± 0.8	NS	2.9 ± 0.6	3.2 ± 0.9	NS
apoB/apoA	0.6 ± 0.1	0.6 ± 0.2	NS	0.6 ± 0.1	0.6 ± 0.2	NS
BMI	22.1 ± 3.9	22.2 ± 3.8	NS	21.5 ± 3.4	22.6 ± 4.0	NS

Values are expressed as mean ± standard deviation, otherwise stated.

*P *value from ANOVA, adjusted for mass significance.

TC indicates total cholesterol; HDLc, high density liprotein cholesterol; LDLc, low density lipoprotein cholesterol; TG, triglycerides; Apo, apolipoprotein; Lp(a), lipoprotein (a); NS, not significant

*Analyses were performed on log transformed data, but non-transformed data are presented in the table.

The distribution of the PPARγ2 genotypes did not differ between males and females (Pro12/Pro, 85 and 82% in males and females, respectively; Pro12/Ala, 15 and 18%, in males and females, respectively). No differences were found between the Pro12/Pro and Pro12/Ala12 genotypes in any of the lipid parameters studied, nor in BMI (data not shown).

There were no differences between all the genotypes in birth weight in males and females. The outcome did not change when all the analyses were adjusted for age and/or pubertal development.

### Birth weight, lipids and genotype interactions

Low birth weight was associated with higher levels of TC, LDLc, apoB, Lp(a), TC-HDLc, TC/HDLc and apoB/apoA in males with the APOE ε3ε4 genotype, after controlling for age, pubertal status, socioeconomic status, gestational age, BMI and cardiorespiratory fitness (Table 5). Low birth weight was also associated with lower levels of HDLc and apoA in males with the APOE ε3ε3 genotype. In females with the APOE ε3ε4 genotype, low birth weight was associated with lower levels of HDLc and higher levels of TG. Low birth weight was not significantly associated with any of the lipid parameters studied in those adolescents with the APOE ε2ε3 or in females with the ε3ε3 genotype.

**Table 5:** Regression coefficients ($\hat{\beta }$) showing the association between birth weight score and lipid and lipoprotein levels by APOE genotypes and sex after controlling for age, pubertal stage, socioeconomic status, gestational age, body mass index and cardiorespiratory fitness

	**Males (n = 260)**						**Females (n = 242)**					
	**ε2/ε3**		**ε3/ε3**		**ε3/ε4**		**ε2/ε3**		**ε3/ε3**		**ε3/ε4**	
	$\hat{\beta }$	**P value**	$\hat{\beta }$	**P value**	$\hat{\beta }$	**P value**	$\hat{\beta }$	**P value**	$\hat{\beta }$	**P value**	$\hat{\beta }$	**P value**

TC*	0.019	0.976	0.198	0.065	-0.401	**0.042**	0.787	0.353	0.157	0.197	-0.313	0.262
HDLc*	0.487	0.242	0.285	**0.011**	0.262	0.238	0.797	0.427	0.153	0.195	0.525	**0.027**
LDLc*	-0.109	0.787	0.121	0.260	-0.622	**0.003**	0.458	0.566	0.116	0.354	-0.302	0.264
TG*	-0.023	0.956	0.001	0.988	0.048	0.824	0.610	**0.031**	-0.153	0.171	-0.477	**0.039**
apoA*	0.424	0.326	0.236	**0.022**	0.328	0.125	0.698	0.314	0.150	0.900	-0.267	0.278
apoB*	-0.092	0.820	0.160	0.145	-0.554	**0.008**	0.428	0.540	-0.172	0.172	-0.076	0.780
Lp(a)*	-0.064	0.863	0.122	0.259	-0.625	**0.002**	-0.301	0.743	0.054	0.667	0.054	0.843
TC-HDLc	-0.019	0.663	0.127	0.227	-0.454	**0.024**	0.518	0.522	0.085	0.501	-0.060	0.816
TC/HDLc	-0.402	0.286	0.014	0.889	-0.406	**0.028**	0.106	0.900	-0.041	0.742	0.243	0.291
apoB/apoA	-0.333	0.387	-0.007	0.946	-0.518	**0.011**	0.142	0.850	0.047	0.699	0.130	0.588

TC indicates total cholesterol; HDLc, high density liprotein cholesterol; LDLc, low density lipoprotein cholesterol; TG, triglycerides; Apo, apolipoprotein; Lp(a), lipoprotein a.

*Analyses were performed on log transformed data.

Low birth weight was associated with lower levels of apoA in males with the APOC3 S1/S1 genotype, and higher levels of Lp(a) in those with both the APOC3 S1/S1 and the S1/S2 genotype. In females, low birth weight was not significantly associated with blood lipids in any of the APOC3 genotypes (Table 6). There were no associations between low birth weight and blood lipids in any of the PPARγ2 genotypes (data not shown). The outcome did not change when BMI was not included in the model. The results did not materially change either when another anthropometric index (i.e. sum of six skinfold thicknesses, percentage of body fat, fat free mass, waist circumference, height squared) rather than BMI was included in the model.

**Table 6:** Regression coefficients ($\hat{\beta }$) showing the association between birth weight score and lipid and lipoprotein levels by APOC3 genotypes and sex after controlling for age, pubertal stage, socioeconomic status, gestational age, body mass index and cardiorespiratory fitness

	**Males (n = 260)**				**Females (n = 242)**			
	**S1/S1**		**S1/S2**		**S1/S1**		**S1/S2**	
	
	$\hat{\beta }$	***P *value**	$\hat{\beta }$	***P *value**	$\hat{\beta }$	***P *value**	$\hat{\beta }$	***P *value**
TC*	0.050	0.609	-0.301	0.211	0.032	0.795	-0.309	0.535
HDLc*	0.184	0.053	-0.148	0.493	-0.063	0.590	0.363	0.346
LDLc*	-0.089	0.360	-0.208	0.387	-0.002	0.987	-0.305	0.528
TG*	0.048	0.616	-0.190	0.405	0.188	0.113	-0.588	0.227
apoA*	0.244	**0.010**	-0.146	0.522	-0.120	0.279	0.281	0.456
apoB*	-0.043	0.668	-0.267	0.268	0.022	0.859	-0.330	0.469
Lp(a)*	-0.294	**0.003**	-0.731	**<0.001**	0.021	0.865	0.020	0.867
TC-HDLc	0.022	0.871	-0.278	0.277	0.050	0.682	-0.409	0.394
TC/HDLc	-0.086	0.355	-0.087	0.722	0.097	0.407	-0.433	0.338
apoB/apoA	-0.144	0.130	-0.166	0.504	0.108	0.351	-0.377	0.367

TC indicates total cholesterol; HDLc, high density liprotein cholesterol; LDLc, low density lipoprotein cholesterol; TG, triglycerides; Apo, apolipoprotein; Lp(a), lipoprotein a.

*Analyses were performed on log transformed data.

## Discussion

The results of the present study show that low birth weight seems to have a deleterious influence on blood lipid profile, particularly in those adolescents with the APOE ε3/ε4 genotype. The results also suggest that low birth weight is associated with lower levels of HDLc and apoA, and higher TC/HDLc and apoB/apoA ratios in male adolescents. Yet, low birth weight is not associated with blood lipid levels in female adolescents. Out of the three genes analyzed, the APOE genotypes seem to be a major determinant factor of lipid and lipoprotein profile in both sexes. Taken together, these results support the notion that intrauterine environment interact with genes in determining blood lipid profile in later life. One possible explanation to these findings is that a reduced foetal growth may have negative consequences on the liver growth. A poor liver growth may cause a down-regulation of the hepatic receptors, as well as disturbances in the synthesis of cholesterol. Consequently, the influence of the APOE genotypes on the lipid and lipoprotein metabolism would be intensified in those individuals with a low birth weight.

The potential underlying mechanism linking low birth weight with and adverse lipid profile in later life remains unclear. Barker et al. [7] suggested that raised serum TC, LDLc, and apoB levels in adult life might be associated with impaired growth during late gestation, when fetal undernutrition has a disproportionate effect on liver growth. Therefore, as the liver regulates lipid metabolism, impaired in utero growth of the liver may program a more adverse lipid profile.

Interpretation of the birth weight effect on blood lipids after adjustment for current body size is controversial, and available results indicate that postnatal changes in size, rather than fetal growth, is important [35]. Our findings show that the influence of low birth weight on HDLc, apoA, TC/HDLc and apoB/apoA in males was not altered after controlling for any of the measures of current body size or by others potential confounders such as age [15], pubertal maturation [27], cardiorespiratory fitness [14,36] or socioeconomic status [37]. It is noteworthy that the associations between birth weight and blood lipids were mainly observed in males. A number of studies have found evidences for a sex related difference in the programming effect of low birth weight and later cardiovascular risk factors [3,38]. Sex related differences in the association between fetal growth and later coronary heart disease risk suggest that male fetuses are more vulnerable to the effects of fetal undernutrition [38]. One of the explanations lays on the fact that male fetuses seem to grow at a faster rate than female foetuses [39,40]. Therefore, assuming that fetal nutritional programming is the primary pathway for the association between birth weight and later cardiovascular disease risk factors it could be expected these associations to be stronger in males compared to females [39,40]. Our results concur with those showed in a meta-analyses by Lawlor et al. [38]. They reported that the association between birth weight and total cholesterol was stronger in males compared to females. The biological explanation for the different outcomes obtained in males and females warrant further investigation. It has been reported that the influence of sex on genes involved in the lipid and glucose metabolism could be attributed to hormonal differences [41,42]. However, we did not measure sex hormones, which hamper a further study of hormone-lipoprotein relationships in the studied population.

Variation in blood lipid levels seems to be partially determined by the APOE genotypes [10], which agree with our data. APOE genotype has also been shown to affect the association between birth weight and blood lipids in children [10]. Garces et al. reported that a greater effect of the APOE genotype on TC, LDLc and apoB levels was found in children with low birth weight [10]. In our study, low birth weight was associated with higher levels of TC and LDLc, apoB, and Lp(a) in males with the APOE ε3/ε4 genotype. Likewise, low birth weight was also associated with higher apoB/apoA ratio, which is a strong cardiovascular risk factor [43]. This observation suggests that the interaction of APOE genotype and birth weight might be an important determinant for future atherosclerosis.

Female adolescents with the APOC3 S1/S2 genotype had elevated TC and non-HDLc levels, which is in agreement with another study [44]. The levels of triglyceride were similar between S1/S1 and S1/S2 genotypes in both males and females as reported in other population samples [45,46]. We also found that low birth weight was associated with lower levels of apoA in males with the APOC3 S1/S1 genotype, whereas low birth weight was associated with higher levels of Lp(a) in males with both S1/S1 and S1/S2 genotypes. This is the first study examining the influence of this polymorphism on the association between low birth weight and blood lipid levels in adolescents.

The PPARγ2 gene plays an important role in the regulation of glucose, lipid and energy metabolism [47], therefore it would be expected that the PPARγ2 genotypes could affect circulating lipid levels. The findings concerning the associations between PPARγ2 genotypes and lipid levels are contradictory [11,41,48-50]. We did not find differences on blood lipid and lipoprotein levels between genotypes, nor did on birth weight. Pfab et al. reported that neither the fetal nor the maternal PPARγ2 genotypes affected the birth weight of the 1950 newborn studied [50]. There is one study underlying the influence of PPARγ2 genotypes on the association between birth weight and lipid levels among elderly people [11]. It was reported that the Ala12 allele was associated with an increased TC, LDLc, and non-HDLc levels only among those who had birth weights lower than 3000 g. We did not find a significant effect of birth weight on lipid levels regarding the analyzed PPARγ2 genotypes, which agree with others [50].

Other factors such as current dietary intake may have affected the lipid levels, but such data were not available in the AVENA Study. There is controversy about the influence of age at the onset of menses on lipid and lipoprotein levels. Yet, no effect of age of menarche on lipid levels have been observed in the AVENA Study population [15]. The inclusion of age of menarche in the analysis rather than pubertal status did not alter the outcome. Birth weight might be a crude marker of the influence of maternal environment on fetal development and therefore underestimate the influence of such factors on offspring health outcomes. Future studies should directly measure potentially modifiable factors such as maternal nutrition, smoking, alcohol consumption or exercise, and relate these to offspring cardiovascular disease risk factors. It also remains possible that other birth measures including ponderal index, head circumference of waist circumference could be more important, but these measures were not available in the present study. The results should be interpreted with caution due to the small sample sizes.

## Conclusion

The results of this study suggest that intrauterine environment interact with the genetic background affecting the lipid profile in later life. The findings indicate that low birth weight has a deleterious influence on lipid profile particularly in adolescents with the APOE ε3/ε4 genotype.

## Competing interests

The authors declare that they have no competing interests. No benefits in any form have been received or will be received from a commercial party related directly or indirectly to the subject of this article.

## Authors' contributions

JRR conceived the hypothesis, conducted the statistical analyses, and drafted the manuscript. IL, FOP and LAM critically revised the drafted manuscript. DGL, AM, MGF, JAM performed the genetic analysis. All authors contributed to the interpretation and discussion of the results, and critically revised the drafted manuscript.

## Note

*AVENA Study Group

**Coordinator: **A Marcos, Madrid.

Local coordinators:

MJ Castillo, Granada.

A Marcos, Madrid.

S Zamora, Murcia.

M García Fuentes, Santander.

M Bueno, Zaragoza, Spain.

***Granada: ***MJ Castillo, MD Cano, R Sola *(Biochemistry)*; A Gutiérrez, JL Mesa, JR Ruiz *(Physical fitness)*; M Delgado, P Tercedor, P Chillón *(Physical activity)*, M Martín, F Carreño, FB Ortega, GV Rodríguez, R Castillo, F Arellano (*Collaborators*). Universidad de Granada. E-18071 Granada.

***Madrid***: A Marcos, M González-Gross, J Wärnberg, S Medina, F Sánchez Muniz, E Nova, A Montero, B de la Rosa, S Gómez, S Samartín, J Romeo, R Álvarez, (*Coordination, immunology*) A Álvarez (*Cytometric analysis*) L Barrios (*Statistical analysis*) A Leyva, B Payá (*Psychological assessment*). L Martínez, E Ramos, R Ortiz, A Urzanqui. *(Collaborators)*. Instituto de Nutrición y Bromatología. Consejo Superior de Investigaciones Científicas (CSIC). E-28040 Madrid.

***Murcia***: S Zamora, M Garaulet, F Pérez-Llamas, JC Baraza, JF Marín, F Pérez de Heredia, MA Fernández, C González, R García, C Torralba, E Donat, E Morales, MD García, JA Martínez, JJ Hernández, A Asensio, FJ Plaza, MJ López (*Diet analysis*). Dpto. Fisiología. Universidad de Murcia. E-30100 Murcia.

***Santander***: M García Fuentes, D González-Lamuño, P de Rufino, R Pérez-Prieto, D Fernández, T Amigo (*Genetic study)*. Dpto. Pediatría. Universidad de Cantabria. E – 19003 Santander.

***Zaragoza***: M Bueno, LA Moreno, A Sarriá, J Fleta, G Rodríguez, CM Gil, MI Mesana, JA Casajús, Vicente Blay, María Guadalupe Blay. (*Anthropometric assessment*). Escuela Universitaria de Ciencias de la Salud. Universidad de Zaragoza. E-50009 Zaragoza.

## Pre-publication history

The pre-publication history for this paper can be accessed here:


